# Antimicrobial susceptibilities and mechanisms of resistance of commensal and invasive *Mycoplasma salivarium* isolates

**DOI:** 10.3389/fmicb.2022.914464

**Published:** 2022-08-01

**Authors:** Li Xiao, Arthur H. Totten, Donna M. Crabb, Thomas Prescott Atkinson, Ken B. Waites

**Affiliations:** ^1^Department of Medicine, University of Alabama at Birmingham, Birmingham, AL, United States; ^2^Department of Pediatrics, University of Alabama at Birmingham, Birmingham, AL, United States; ^3^Department of Pathology, University of Alabama at Birmingham, Birmingham, AL, United States

**Keywords:** *Mycoplasma salivarium*, susceptibility, antimicrobial resistance, mutation, minimum inhibition concentration, quinolone resistance determining region, Clinical and Laboratory Standards Institute, single nucleotide polymorphisms

## Abstract

*Mycoplasma salivarium*, an oral commensal organism, can cause severe invasive infections in immunocompromised individuals. Currently there is no treatment guidance for such infections. We performed antimicrobial susceptibility tests on 39 commensal and invasive *M. salivarium* isolates and investigated the mechanisms of antimicrobial resistance. Clindamycin was the most active agent [minimum inhibition concentration (MIC) range: 0.004–128 mg/L, MIC_50_ = 0.031 mg/L, MIC_90_ = 0.125 mg/ml], followed by tetracycline and levofloxacin. All isolates were resistant to erythromycin (MIC ≥4 mg/L) due to the presence of 2057A (*Escherichia coli* numbering) in 23S rRNA. Three isolates with elevated clindamycin MICs (≥8 mg/L) harbored A2058T/G mutations in *23S rRNA* gene; four sequential isolates from one patient developed C2611T and A2059G mutations accompanying the increase of clindamycin MICs. Five isolates with elevated tetracycline MICs (≥4 mg/L) had mutations in *16S rRNA* gene (A965G/T, G966T, or A967C/T) and one of them harbored *TetM*. Nine isolates with elevated levofloxacin MICs (≥4 mg/L) had one or more mutations in *gyrA*, *gyrB*, *parC*, or *parE*. Susceptibility breakpoints for clindamycin, tetracycline and levofloxacin were suggested to be ≤0.125, ≤2, and ≤2 mg/L, respectively. Antimicrobial resistance to any of the three agents (clindamycin, tetracycline, or levofloxacin) was documented in 12 (34.3%) non-duplicate isolates, of which 10 were invasive. Levofloxacin resistance was most frequent (25.7%). Multi-drug resistance was also observed (14.3%). This study demonstrates the frequent occurrence of antimicrobial resistance in *M. salivarium*, emphasizing the need for culture and susceptibility testing to guide antimicrobial therapy.

## Introduction

*Mycoplasma salivarium* is one of several mycoplasmal species that are endogenous within the human oropharynx, usually as commensals. This organism commonly resides in dental plaque and gingival sulci and has been associated with periodontal disease (Uchida et al., 1981; Watanabe et al., 1986; Lamster et al., 1997). It is also reported to be heavily associated with oral leukoplakia (Mizuki et al., 2017) and oral carcinoma in patients with Fanconi anemia (Henrich et al., 2014). Extra-oropharyngeal infections are uncommon, but this organism has been detected in synovial fluid in persons with acute or chronic arthritis, prosthetic joint infections, empyemas, abscesses in the brain, and infections in other body sites (So et al., 1983; Johnson et al., 2007; Grisold et al., 2008; Orsted et al., 2011; Baracaldo et al., 2012; Buchsel et al., 2016; Thoendel et al., 2017; Totten et al., 2021). A recent study showed that lower airway enrichment of *M. salivarium* is associated with poor clinical outcome in mechanically ventilated COVID-19 patients (Sulaiman et al., 2021). Invasive infections in a normally sterile body site most often occurs in association with humoral immune deficiency or other immunosuppressed states (So et al., 1983; Buchsel et al., 2016; Thoendel et al., 2017; Totten et al., 2021). Currently there is no *M. salivarium* infection prevalence data among immunocompromised populations.

Besides a few case reports, very little data regarding what constitutes the most effective treatments or *in vitro* antimicrobial susceptibility profiles of *M. salivarium* are available in published literature (Grisold et al., 2008; Buchsel et al., 2016; Totten et al., 2021). Treatment alternatives are somewhat limited, as all *Mycoplasma* spp. are intrinsically resistant to β-lactams due to their lack of a cell wall, but they are generally susceptible to fluoroquinolones and tetracyclines, as well as macrolides and/or lincosamides (Waites et al., 2014). There are only three case reports performed *in vitro* susceptibility testing for *M. salivarium* and the main drugs showing *in vitro* susceptibility are clindamycin, tetracycline, and moxifloxacin (Grisold et al., 2008; Buchsel et al., 2016; Totten et al., 2021). Acquired resistance to macrolides, lincosamides, fluoroquinolones, and/or tetracyclines has been documented in all of the *Mycoplasma* spp. known to be pathogenic in humans (Waites et al., 2014). There are two reports described fluoroquinolone resistance in invasive *M. salivarium* isolates causing septic arthritis and another report detected a mutation possibly related to macrolide resistance in *M. salivarium* that caused prosthetic joint infection (Buchsel et al., 2016; Thoendel et al., 2017; Totten et al., 2021). In this study, we determined the minimum inhibition concentrations (MICs) for four classes of antimicrobial agents against clinical isolates and reference strains of *M. salivarium* and investigated the genetic mechanisms associated with antimicrobial resistance. This study provides guidance for empiric treatment of invasive *M. salivarium* infections and determines the extent that acquired antimicrobial resistance occurs.

## Materials and methods

### Microorganisms

A total of 39 *M. salivarium* isolates were evaluated (Supplementary Table S1). Among them, two were ATCC strains, 33 were clinical isolates from non-duplicate patients collected from 13 facilities in 12 states (AL, CO, CT, IL, KS, MA, MN, OH, PA, TN, UT, and WA) between 2002 and 2021, and four were additional isolates obtained sequentially from synovial fluid of one of the above non-duplicate patients between 2018 and 2021. Among the 33 non-duplicated clinical isolates, 13 were commensals from throats of healthy adults in 2013 in a previous study (Centor et al., 2015), 20 were invasive isolates from synovial fluid (5), lower respiratory tract (5), chest wall/sternum (3), pleural fluid (2), or miscellaneous tissues/fluid (5). All clinical isolates were obtained by culture in SP4 broth incubated at 37°C for 3–5 days, and their species identities were confirmed by sequencing of the full length *16S rRNA* gene using primer pair fD1 and rP1 (Weisburg et al., 1991; La Scola et al., 1997).

### Antimicrobial susceptibility test

Antimicrobial susceptibility of the 39 isolates against levofloxacin, clindamycin, erythromycin, and tetracycline were determined by broth microdilution using SP4 broth in accordance with methods established for human mycoplasmas by the Clinical and Laboratory Standards Institute (2011). Considering the close phylogenetic relationship, *Mycoplasma hominis* strain ATCC 23114 was used for quality control. The MIC plates were sealed with Parafilm and incubated at 37°C for 72–120 h until the color of growth controls changed which indicating the time point to read the MICs. MICs for levofloxacin, clindamycin, and tetracycline were initially interpreted using susceptibility breakpoints published for *M. hominis* (Clinical and Laboratory Standards Institute, 2011). Erythromycin MICs were initially interpreted using the breakpoint for *Mycoplasma pneumoniae* as there is no erythromycin breakpoint established by the Clinical and Laboratory Standards Institute (CLSI) for *M. hominis* (Clinical and Laboratory Standards Institute, 2011).

### Genetic analyses

PCR and sequencing primers were designed or obtained from published literature for designated targets (Supplementary Table S2). According to the genome sequence of ATCC strain 23064 (GenBank number NZ_LR214938), *M. salivarium* has a single copy of *16S rRNA* and *23S rRNA* genes. Erythromycin and clindamycin resistance mechanisms were investigated by sequencing the full length of the *23S rRNA* gene and ribosomal protein genes *rplD* (L4) and *rplV* (L22). Quinolone resistance mechanisms were investigated by sequencing the quinolone resistance determining region (QRDR) of DNA gyrase genes *gyrA* and *gyrB* and DNA topoisomerase IV genes *parC* and *parE*. Genetic mechanisms associated with tetracycline resistance were assessed by *16S rRNA* gene sequencing and testing for the presence of the *tetM* gene (Blanchard et al., 1992). Genomic DNA of *Ureaplasma urealyticum* serovar 9 that is known to contain the *tetM* sequence was used as *tertM* PCR positive control. A no template negative control was included in every PCR run. PCRs were performed on a Veriti 96-well thermal cycler (Applied Biosystems, Foster City, CA, United States) with a 25 μl PCR reaction volume containing 0.4 μmol/L of each primer, 2.5 μl of 10X AccuPrime Pfx reaction mix (Thermo Fisher, Fremont, CA, United States), 0.5 U of AccuPrime Pfx DNA polymerase and 2 μl of template DNA. Amplicons were sequenced by Sanger technique at the UAB Heflin Genomics Center and analyzed using CLC Genomics Workbench 21 (Qiagen, Redwood City, CA, United States). *Mycoplasma salivarium* reference sequences were obtained from the genome sequence of *M. salivarium* strain NCTC 10113 (alternative strain name: ATCC 23064, GenBank number NZ_LR214938). Reference sequences for *Escherichia coli* were obtained from the genome sequence of strain K-12, substr. MG1655 (GenBank number U00096.3). Reference sequence for *tetM* was GenBank U08812.1. The assembled sequences were submitted to GenBank and the accession numbers are: OM864597 to OM864634 for*16S rRNA* gene, ON023780 to ON023817 for *23S rRNA* gene, ON036345 to ON036382 for *rplD*, ON036383 to ON036420 for *rplV*, ON036193 to ON036230 for *gyrA*, ON036231 to ON036268 for *gyrB*, ON036269 to ON036306 for *parC* and ON036307 to ON036344 for *parE*.

### Statistical analysis

Chi-square analysis or Fisher’s exact test was performed to compare the antimicrobial resistance rates between invasive and non-invasive isolates using IBM SPSS 27 (IBM Corp., Armonk, NY, United States). A resistant isolate is defined as an isolate is resistant to any of the three drugs excluding erythromycin. If an isolate is resistant to two or more drugs, it is considered multi-drug resistant. A value of *p* < 0.05 was considered statistically significant.

## Results

### Erythromycin MICs and resistance associated mutations

**Figure 1:**
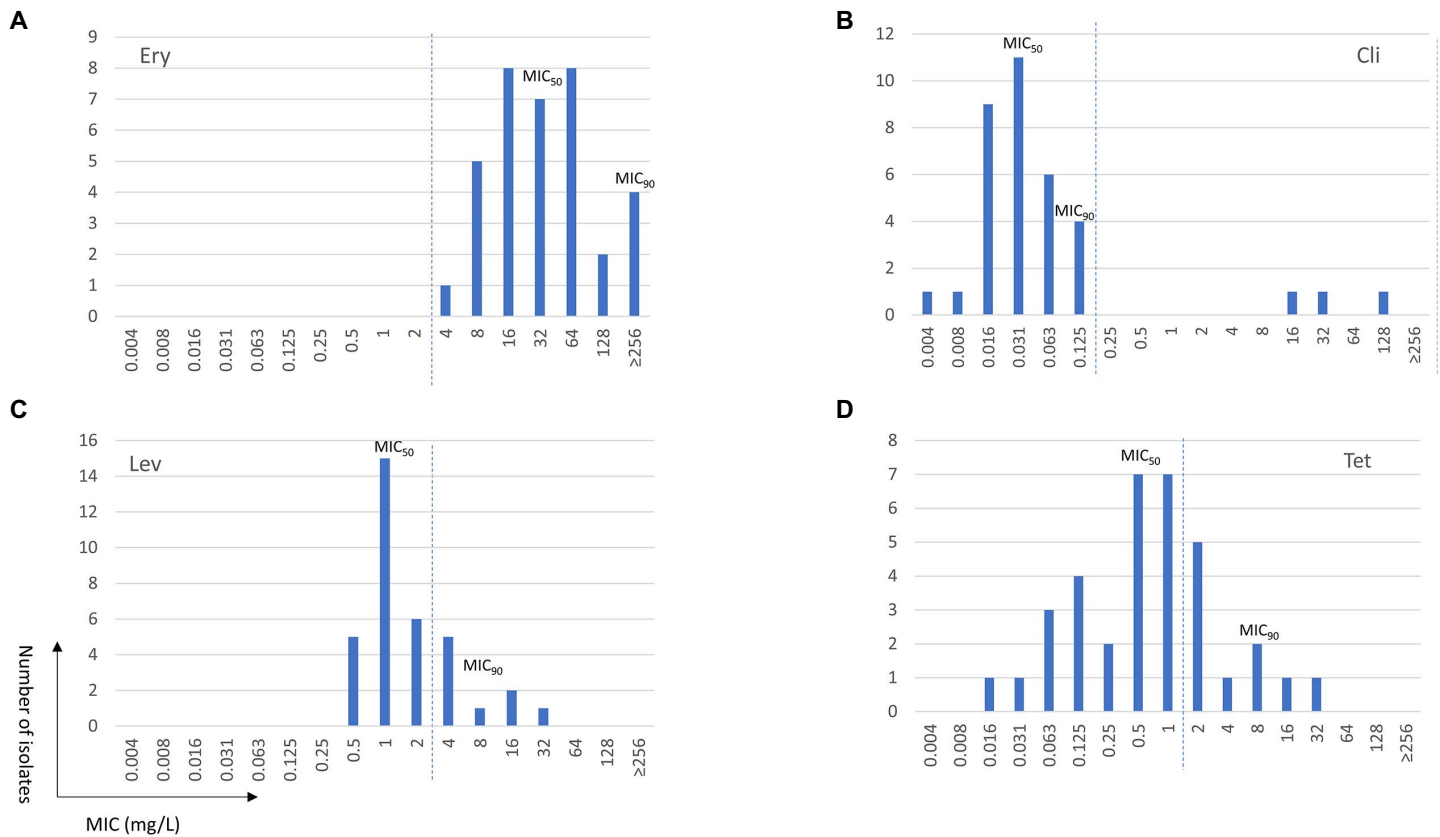
Minimum inhibition concentration (MIC) distributions of erythromycin (Ery, **A**), clindamycin (Cli, **B**), levofloxacin (Lev, **C**), and tetracycline (Tet, **D**) for *Mycoplasma salivarium* isolates. The dashed line divides the cutoff values for wild type (left) and mutant (right) strains.

A summary of MIC data on 35 non-duplicated isolates (two ATCC strains and 33 non-duplicated clinical strains) is provided in Table 1, and a complete tabulation of MICs and genotypic analyses for the 35 *M. salivarium* isolates tested is provided in Supplementary Table S1. The distribution of MIC data is shown in Figure 1.

**Table 1:** Minimum inhibition concentration summary for 35 *M. salivarium* Isolates.

Reagent	Suggested susceptibility breakpoint (mg/L)	MIC Range (mg/L)			MIC_50_ (mg/L)			MIC_90_ (mg/L)			No. and percentage resistant^a^			
		Overall (*n* = 35)	Non-invasive (*n* = 15)	Invasive (*n* = 20)	Overall (*n* = 35)	Non-invasive (*n* = 15)	Invasive (*n* = 20)	Overall (*n* = 35)	Non-invasive (*n* = 15)	Invasive (*n* = 20)	Overall (*n* = 35)	Non-invasive (*n* = 15)	Invasive (*n* = 20)	*p*-value
Levofloxacin	≤2	0.5–32	0.5–2	0.5–32	1	1	2	8	2	16	9 (25.7)	0 (0.0)	9 (45.0)	0.004
Tetracycline	≤2	0.016–32	0.25–32	0.016–16	0.5	1	0.25	8	2	8	5 (14.3)	1 (6.7)	4 (20.0)	0.356
Clindamycin	≤0.125	0.004–128	0.016–32	0.004–128	0.031	0.031	0.031	0.125	0.125	0.063	3 (8.6)	1 (6.7)	2 (10.0)	1
Erythromycin	≤0.5 (Mpn)	4 to ≥256	8 to ≥256	4 to ≥256	32	32	32	≥256	128	≥256	35 (100.0)	15 (100.0)	20 (100.0)	1
Total^b^											12 (34.3)	2 (13.3)	10 (50.0)	0.034

a Susceptibility was designated using suggested MIC breakpoints determined by this study for levofloxacin (≤2 mg/L), tetracycline (≤2 mg/L), and clindamycin (≤0.125 mg/L). The breakpoint for erythromycin was adopted from the Clinical and Laboratory Standards Institute reference for *M. pneumoniae* (Mpn, S ≤ 0.5 mg/L).

b Erythromycin was excluded in overall resistance rate calculation.

Similar to *M. hominis* (Waites et al., 2014), elevated MICs to erythromycin ranging from 4 to ≥256 mg/L were observed in all *M. salivarium* isolates tested (Figure 1A). Four isolates showed highly elevated MICs to erythromycin (≥256 mg/L). Mutations in domain V in 23S rRNA are known to be associated with erythromycin resistance in mycoplasmas (Waites et al., 2014). Using *E. coli 23S rRNA* gene as reference, a G to A transition at position 2057 (*E. coli* numbering) in the central loop of domain V was identified in all isolates. This alteration is reported to be associated with intrinsic resistance to the 14- and 15-membered macrolides in *M. hominis* and several *Mycoplasma* spp. of animal origin (Whithear et al., 1983; Furneri et al., 2000; Pereyre et al., 2002; Stakenborg et al., 2005; Lysnyansky et al., 2015). In the 4 isolates with erythromycin MIC ≥256 mg/L, three harbored mutations at position 2058 (A to T/G) and one had a 9-amino acid deletion at position 139 (Δ139EAKKEAKAT) in ribosomal protein L22 (Table 2). A2058T mutation was also reported in a case report of *M. salivarium* caused prosthetic joint infection without *in vitro* susceptibility data (Thoendel et al., 2017).

**Table 2:** Clindamycin MIC and alterations in *23S rRNA* gene and ribosomal proteins.

Isolate	MIC (mg/L)		Alterations		
	Cli	Ery	*23S rRNA* (*Escherichia coli* numbering)	L4 (Amino acid)	L22 (Amino acid)
ATCC 23064	0.031	16	-	-	-
ATCC 14277	0.031	16	-	-	-
48759	0.004	8	T391C (343); G426A (375), C1260T (1222)	V228I	A202V
73363	0.008	64	T391C (343); G676A (625); C2223T (2207)	-	-
49906	0.016	32	C385T (337); T391C (343);	-	A136T; A144T; A146T; P158S; T186A; T195A; A202V; P213T
59228	0.016	16	T391C (343)	-	-
59258	0.016	32	T391C (343); G2685A (2668)	P21S	V204I
59260	0.016	64	T391C (343)	-	R35T (mixed); A136T; A144T; A146T; P158S; T186A; T195A; A202V
59838	0.016	8	T391C (343); C945T (893)	-	A136T; A144T; A146T; P158S; T186A; T195A; A202V
62771	0.016	128	T391C (343); G1364A (1328)	-	-
83426	0.016	32	T391C (343); G1130A (1080); C1890T (1874)	-	T195A; A202V; D224N
83735	0.016	16	T391C (343); G775A (723)	-	P158S
84893	0.016	64	T391C (343); T2027C (2011)	P21S	E143G
59229	0.031	16	T391C (343)	P21S	-
59262	0.031	16	T391C (343); G2388A (2371)	-	-
59263	0.031	32	T391C (343); G428A (377)	N134D	K199T; E200K
59266	0.031	128	T391C (343); G650A (602)	-	-
59272	0.031	16	T391C (343); G1165A (1115); G1354A (1318)	-	-
72114	0.031	8	T391C (343); C1277T (1239)	-	-
82217	0.031	4	T391C (343); G428A (377)	A233T	A155V; T186A
84726	0.031	64	T391C (343)	-	-
84850	0.031	32	T391C (343); A500G (449); G650A (602); T1318C (1282); T1753C (1712)	-	A136T; A144T; A146T; P158S; T186A; T195A; A202V
63019	0.063	16	-	-	D123N
72036	0.063	64	T391C (343); C1277T (1239)	-	F93L
78192	0.063	32	T391C (343)	A32V	S150T
81314	0.063	64	T391C (343)	H164Y	-
82746	0.063	256	T391C (343)	-	Δ139EAKKEAKAT; A202T
84114	0.063	8	C140T (139); T391C (343); G606A (558); G650A (602); G2172A (2156)	-	-
59240	0.125	8	C385T (337); T391C (343); G1614A (1573)	N134D	-
59243	0.125	64	A340G (insertion 293); T391C (343)	P21S	A136T; A202T
59247	0.125	64	C140T (139); T391C (343); C2841T (2841)	-	-
59271	0.125	32	C590T (540)	-	-
72904*	0.125	8	T391C (343); C1277T (1239); **C2628T (2611)**	-	-
85051*	4	128	T391C (343); C1277T (1239); **A2075G (2059)**; **C2628T (2611)**	-	-
80422*	8	32	T391C (343); C1277T (1239); **A2075G (2059)**; **C2628T (2611)**	-	-
82061*	8	256	T391C (343); C1277T (1239); **A2075G (2059)**; **C2628T (2611)**	-	-
69499	16	256	T391C (343); G1186A (1136); C1351T (1315); G1373A (1337); **A2074G (2058)**	P21S	-
59283	32	256	T391C (343); G1471A (1436); **A2074G (2058)**	T5I; A32V	P158S; T186A; T195A; A202V; D224N
73273	128	256	T391C (343); G650A (602); C776T (724); **A2074T (2058)**; C2423T (2406)	-	-

1. Both ATCC strains 23064 and 14277 were used as reference. 2. All isolates have G2057A transition compared to *E. coli*. 3. * Sequencial isolates from the same patient after isolate 72114. 4. Mutations associated with resistance were bolded. Cli, clindamycin and Ery, erythromycin.

### Clindamycin MICs and resistance associated mutations

The MIC range for clindamycin in the 35 non-duplicated isolates was 0.004–128 mg/L. The MIC distribution showed a bimodal format (Table 1; Figure 1B). Using the CLSI susceptibility breakpoint for *M. hominis*, 32 out of 35 (91.4%) isolates were susceptible with MICs ≤0.125 mg/L, while the other 3 isolates had elevated MICs ranging from 16 to 128 mg/L. The three isolates were obtained between 2013 and 2018 from throat swab of a healthy volunteer and synovial fluid and sputum of two other patients. These isolates were also highly resistant to erythromycin (MIC ≥256 mg/L). Overall, clindamycin was the most potent drug tested with MIC_50_ and MIC_90_ values of 0.031 and 0.125 mg/L, respectively.

Clindamycin resistance in *M. hominis* has been shown to be related to mutations in 23S rRNA at positions 2059 and 2611 (*E. coli numbering*; Pereyre et al., 2002). In this study, the 23S rRNA mutations were at position 2058 (A to T/G) in all 3 *M. salivarium* isolates with elevated MICs for clindamycin (Table 2). These mutations were not observed in the remaining isolates with MICs ≤0.125 mg/L. Interestingly, the isolate with a 9-amino acid deletion in ribosomal protein L22 that was highly resistant to erythromycin was susceptible to clindamycin. In the sequential isolates collected from one patient between 2018 and 2021, mutations emerging with the development of clindamycin resistance were observed (Supplementary Table S1). The initial isolate (72114) was susceptible to clindamycin (MIC = 0.031 mg/L). Emergence of a C2611T mutation in the second isolate (72094) caused a slight increase of MIC (0.125 mg/L); then the presence of mutation A2059G caused further elevation of MICs to clindamycin (MICs ≥8 mg/L) in all subsequent isolates. There were many other single nucleotide polymorphisms (SNPs) identified in *23S rRNA*, *rplD* (L4) and *rplV* (L22) genes in the clinical strains compared to the two ATCC strains which did not exhibit elevated clindamycin MICs (Table 2; Supplementary Tables S3 and S4).

Considering the MIC distribution and the genetic alterations identified in these isolates, a clindamycin susceptibility breakpoint for *M. salivarium* of 0.125 mg/L, one dilution below to that of *M. hominis* (0.25 mg/L; Clinical and Laboratory Standards Institute, 2011), can be suggested.

### Levofloxacin MICs and resistance associated mutations

The MIC range for levofloxacin was 0.5–32 mg/L and MIC_50_ and MIC_90_ values were 1 and 8 mg/L, respectively, in the 35 non-duplicated isolates (Table 1; Figure 1C). There were 26 out of 35 (74.3%) isolates having MICs ≤2 mg/L, while the remaining nine isolates had elevated MICs ranging from 4 to 32 mg/L (Table 3). These nine isolates were obtained between 2014 and 2021 from synovial fluid, lower respiratory tract, chest wounds, or pelvic aspirate, and all were invasive (Supplementary Table S1).

**Table 3:** Levofloxacin MIC and alterations in GyrA, GryB, ParC, and ParE proteins.

Isolate	MIC (mg/L)	Amino acid alterations (*E. coli* numbering)			
	Lev	GyrA	GyrB	ParC	ParE
ATCC 23064	0.5	-	-	-	-
ATCC 14277	2	-	-	-	-
49906	0.5	-	-	-	-
59240	0.5	-	-	-	-
59266	0.5	-	-	-	-
73363	0.5	-	-	-	-
48759	1	-	-	-	-
59247	1	-	-	-	-
59258	1	-	-	-	-
59260	1	-	-	-	-
59262	1	-	-	-	A411V
59263	1	-	-	-	-
59271	1	-	-	-	-
59272	1	-	-	-	-
59283	1	-	-	-	-
59838	1	-	-	-	-
62771	1	-	-	-	-
72036	1	-	-	-	A411V
81314	1	-	-	-	-
82217	1	-	-	-	-
83735	1	-	-	-	-
59229	2	-	-	-	-
59243	2	-	-	-	-
59228	2	-	-	-	A411V
69499	2	-	-	-	-
84726	2	-	-	-	A411V
63019	4	S132A	-	-	A411V; **D420N**
78192	4	-	-	-	**D420N**
82746	4	-	-	**E84K**	-
83426	4	-	-	P59S; A144T	A411V
84114	4	-	D409N	E151K	-
84893	8	-	-	-	A411V; **D420N**
85051^*^	8	S84P	-	**E84K**	A411V
72904^*^	16	S84P	-	**E84K**	A411V
73273	16	-	-	-	**D420N**
80422^*^	16	S84P	-	**E84K**	A411V
82061^*^	16	S84P	-	**E84K**	A411V
84850	16	S84P	-	**S80I**	-
72114	32	S84P	-	**E84K**	A411V

Both ATCC strains 23064 and 14277 are used as reference. Mutations previously reported to be associated with resistance are bolded. Lev, levofloxacin.

* Sequencial isolates from the same patient after isolate 72114.

Fluoroquinolone resistance in *M. hominis* is associated with mutations in the QRDR of *gyrA*, *gyr B*, *parC*, or *parE* genes (Waites et al., 2014). The nine isolates with elevated MICs had one or more mutations in these genes (Table 3). Among them, three had mutations in ParC (S80I and E84K, *E. coli* numbering) and 4 had mutation in ParE (D420N, *E. coli* numbering). These mutations have been reported to be associated with fluoroquinolone resistance in *M. hominis*, *M. genitalium*, and/or *U. urealyticum* (Bebear et al., 2000; Tagg et al., 2013). Six isolates had multiple mutations. GyrA mutation S84P appeared together with ParC mutations S80I/E84K in one non-duplicate isolate and the five isolates from one patient that had a higher levofloxacin MIC (≥8 mg/ml). In the 26 isolates with levofloxacin MIC ≤2 mg/L, 21 had no mutations and four had single mutation (A411V in ParE) outside the QRDR, which is unlikely to be associated with quinolone resistance. The complete list of SNPs identified are summarized in Supplementary Table S5. Thus, the cutoff of levofloxacin susceptibility breakpoint could tentatively be set at ≤2 mg/L, similar to other human *Mycoplasma* spp. (Clinical and Laboratory Standards Institute, 2011).

### Tetracycline MICs and resistance associated mechanisms

The MIC range for tetracycline was 0.016–32 mg/L in the 35 non-duplicated isolates (Table 1; Figure 1D). There were five isolates (four invasive and one commensal) having elevated tetracycline MICs of 4–32 mg/L, while others were ≤2 mg/L (Table 4; Supplementary Table S1).

**Table 4:** Tetracycline MIC and genetic alterations.

Isolate	Tet MIC (mg/L)	TetM	*16S rRNA* gene alteration (*E. coli* numbering)
ATCC 23064	0.5	-	-
ATCC 14277	1	-	-
63019	0.016	-	G974T (1023)
49906	0.031	-	C936T (986); A1355G (1408)
73273	0.063	Pos	C223T (264); C368T (409)
73363	0.063	Pos	-
83735	0.063	-	G985A (1039)
62771	0.125	-	-
69499	0.125	-	-
78192	0.125	-	-
81314	0.125	-	-
59240	0.25	Pos	-
72036	0.25	-	-
48759	0.5	-	C585T (625)
59228	0.5	Pos	-
59262	0.5	-	-
59263	0.5	Pos	G984A (1038); G1213T (1267)
59272	0.5	Pos	-
59838	0.5	-	-
59229	1	Pos	-
59247	1	Pos	-
59266	1	Pos	-
59271	1	-	-
82217	1	-	C1396T (1449)
84893	1	-	-
59258	2	Pos	G224A (265)
59260	2	Pos	C304T (345); **A915G (mixed, 965)**; G923A (973)
59283	2	Pos	**A915G (965)**
82746	2	-	-
84726	2	-	-
72904^*^	4	-	**A915T (965)**; C1141T (1195)
80422^*^	4	-	**A915T (965)**; C1141T (1195)
82061^*^	4	-	**A915T (965)**; C1141T (1195)
83426	4	-	G376A (417); **A915G (965)**
85051^*^	4	-	**A915T (965)**; C1141T (1195)
72114	8	-	**A915T (965)**; A919C (969); C1141T (1195)
84850	8	-	T6C (41); **A915G (965)**; **A917T (967)**; A1406G (1462)
84114	16	-	G659A (519); **G916T (966)**; **A917C (967)**
59243	32	Pos	**A915G (965)**; G1213T (1267)

Both ATCC strains 23064 and 14277 are used as reference. Mutations known to be associated with resistance are bolded. Tet, tetracycline.

* Sequential isolates from the same patient after isolate 72114.

**Figure 2:**
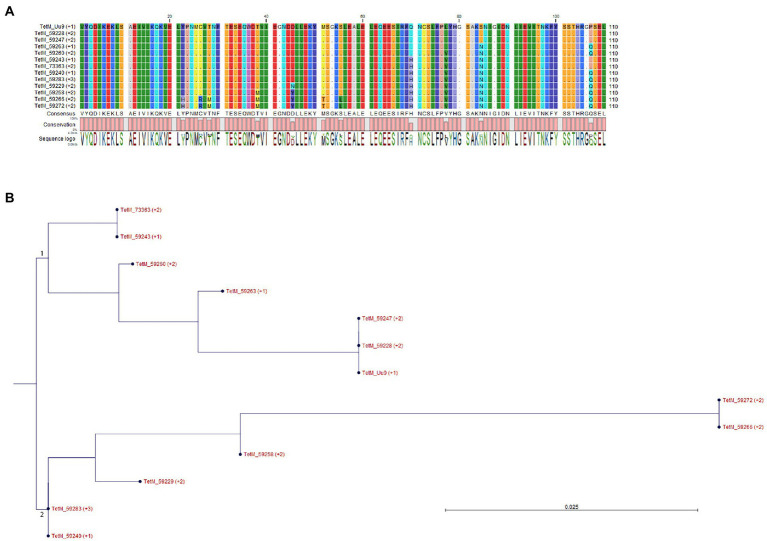
Alignment and grouping of the derived partial amino acid sequences of TetM. **(A)** Sequence alignment. Residues identical to that of TetM from *Ureaplasma urealyticum* serovar 9 (TetM_Uu9) were shown as dot. **(B)** Phylogenetic tree was constructed based on the alignment of **(A)** by Neighbor Joining method using Jukes–Cantor protein distance measurement and bootstrap of 100 replicates. The major groups 1 and 2 were indicated.

All five isolates with elevated tetracycline MICs had mutations at position 965, 966, or/and 967 in *16S rRNA* gene (Table 4), which have been reported to be associated with tetracycline resistance in *M. hominis* induced by *in vitro* selection (Waites et al., 2014) and in *M. genitalium* clinical specimens with unknown tetracycline exposure history (Le Roy et al., 2021). There were 2 (out of 5) other commensal isolates with MIC of 2 mg/L harboring A965G mutation in*16S rRNA* gene, and one of them was a mix with wild type. Other SNPs identified in the *16S rRNA* gene not associated with tetracycline resistance are listed in Supplementary Table S6. A *tetM* element was detected in 13 isolates (37.1%) and 12 of them had MIC ≤2 mg/L (Table 4; Supplementary Table S1). The majority (10) of the *tetM*-positive isolates were commensals. Sequencing was successful for 12 isolates and derived partial amino acid sequences of TetM can be divided into two major groups and eight different types (Figure 2). BLASTP analysis revealed that group 1 (containing six sequences) was 99%–100% identical to the sequences of TetM of *E. faecium*, *Streptococcus suis*, *Clostridioides difficile* and others, while group 2 (containing another six sequences) was 98%–100% identical to the TetM sequences of *S. pneumoniae*, *E. coli* and others. Expanded comparison with TetM sequences from *Ureaplasma* spp. obtained by Dumke (2021) showed a clear cluster of *M. salivarium* group (containing eight sequences) and an *Ureaplasma* group (containing five sequences from *Ureaplasma* spp. and four sequences from *M. salivarium*), suggesting that these *M. salivarium* isolates had acquired *tetM* element from two major different sources.

We suggest tetracycline susceptibility breakpoint for *M. salivarium* could be tentatively set at ≤2 mg/L, one dilution below that of *M. hominis* (4 mg/L). Having a lower cutoff of ≤1 mg/L for *M. salivarium* may be appropriate, but additional isolates would need to be investigated and treatment outcomes assessed in order to make such a decision with confidence.

### Antimicrobial resistance frequencies

Using the above suggested antimicrobial susceptibility breakpoints for *M. salivarium*, the occurrence of acquired resistance, besides intrinsic erythromycin resistance, to one or more antimicrobial classes was observed in 12 (34.3%) non-duplicate isolates. Among them, nine were resistant to levofloxacin (25.7%), five to tetracycline (14.3%) and three to clindamycin (8.6%; Table 1). Half of the invasive isolates (10/20) were resistant, compared to only two resistant ones in non-invasive isolates (2/15, 13.3%, *p* = 0.034; Table 1). Specifically, for levofloxacin, all non-invasive isolates were susceptible, while 45.0% of invasive isolates (9/20) were resistant (*p* = 0.004, Table 1). Multi-drug resistance was also observed. There were five invasive isolates (5/35, 14.3%) from non-duplicated patients showing resistance to two classes of antimicrobials (Supplementary Table S1). Among those, one was resistant to levofloxacin and clindamycin and four were resistant to levofloxacin and tetracycline. For the sequential isolates, the first two isolates from 2018 were resistant to levofloxacin and tetracycline; the three subsequent isolates from 2019 to 2021 were resistant to all antimicrobials tested. There were no obvious changes of MICs or resistance to clindamycin, levofloxacin and tetracycline among invasive isolates over time.

## Discussion

We have determined the MICs of commensal and invasive *M. salivarium* isolates and suggested the susceptibility breakpoints for clindamycin, tetracycline and levofloxacin. Mutations associated with antimicrobial resistance were identified. To our knowledge, this is the first *in vitro* survey of invasive *M. salivarium* isolates collected over two decades from a broad geographic area of US, comparing reference type strains and throat isolates from healthy volunteers to examine the *in vitro* antimicrobial susceptibilities of this organism.

*Mycoplasma salivarium* is now becoming a recognized cause of opportunistic infections in immunocompromised hosts (So et al., 1983; Buchsel et al., 2016; Thoendel et al., 2017; Totten et al., 2021). Our results have important implications for diagnosis and management of *M. salivarium* infections. Macrolides such as erythromycin are to be avoided since there was universal resistance in all isolates tested. Clinical failure or *in vitro* resistance has already been observed on erythromycin and clarithromycin in previous reports (So et al., 1983; Buchsel et al., 2016; Totten et al., 2021). Acquired resistance to one or more classes of antimicrobials, presumably driven by selective antimicrobial pressure, occurred in half of the invasive isolates tested, and most commonly affected fluoroquinolones, possibly because they are among the most commonly prescribed antimicrobials. Our findings also support the need for attempting isolation of *M. salivarium* by culture whenever a clinically significant infection is suspected and obtaining *in vitro* antimicrobial susceptibilities through a reference laboratory whenever possible for guidance of antimicrobial choices. Based on our data, first line treatments should include clindamycin and tetracycline since the occurrence of resistance was lowest in these agents. It is known that the two drugs are distributed widely to tissues and body fluids, including the invasive infection sites (Baird et al., 1978; Beauduy and Winston, 2017). Previous case reports have shown clinical cure by treatment using clindamycin, doxycycline or minocycline in joint and brain infections (So et al., 1983; Orsted et al., 2011; Baracaldo et al., 2012; Buchsel et al., 2016). Moxifloxacin can be successful if the sensitivity is approved by *in vitro* susceptibility testing (Grisold et al., 2008), even the isolate was resistant to levofloxacin (Totten et al., 2021). New synthetic tetracycline derivatives such as omadacycline and the pleuromutilin lefamulin, both of which have activity *in vitro* against other human mycoplasmas that are resistant to other drug classes (Waites et al., 2016, 2017), may also be useful for treatment of *M. salivarium* infections, but thus far they have not been evaluated against this species.

This study also determined the molecular mechanisms of antimicrobial resistance in *M. salivarium*. The intrinsic erythromycin resistance in *M. salivarium* was due to the presence of 2057A in 23S rRNA, similar in several other *Mycoplasma* species (Whithear et al., 1983; Furneri et al., 2000; Pereyre et al., 2002; Stakenborg et al., 2005; Lysnyansky et al., 2015). In the sequential isolates from the same patient, a gradual increase of erythromycin and clindamycin MICs along with the development of C2611T and A2059G point mutations in 23S rRNA was observed, indicating a rapid evolution/adaptation of this organism. Although no treatment information was available for this patient, macrolide/clindamycin antibiotic pressure might be present during the study period. We also noticed that isolate 80422 collected in 2019 had a lower erythromycin MIC (32 mg/ml) compared to the two later isolates 82061 and 85051 collected in 2020 and 2021 (MIC = 256 and 128 mg/ml), while all three isolates had the same sequences in *23S rRNA*, *rplD* and *rplV*. Other mechanisms of resistance might be involved and worth further investigation. We identified a 9-amino acid deletion at position 139 in ribosomal protein L22 that caused a high MIC for erythromycin (≥256 mg/ml) but not for clindamycin. The amino acid sequence around position 139 is unique for *M. salivarium* and may provide an interacting site specific for erythromycin but not for clindamycin.

We noticed there were 12 (out of 13) isolates harboring *tetM* sequence that were not resistant to tetracycline. Since all isolates were grown directly from original clinical specimens and no filter-clone procedure was performed to purify the organisms, there could be contaminations of *tetM* elements from other organisms carried in the original specimens. On the other hand, *tetM*-positive isolates susceptible to tetracyclines have been observed in *M. hominis* and *Ureaplasma* spp. (Degrange et al., 2008; Beeton et al., 2009). A recent study on *Ureaplasma* isolates showed that less than 10% of strains harboring *tetM* sequences were phenotypically resistant to tetracycline (Dumke, 2021). The diversity of the TetM sequences identified in this study suggests multiple acquisition sources, which means it is unlikely that the majority of the isolates acquired dysfunctional *tetM* genes. Other mechanisms of *tetM* regulation, such as transcriptional attenuation, or regulation mediated by small RNA (Su et al., 1992; Le Neindre et al., 2022), need further investigation. It is possible that tetracycline resistance could be induced through subculture of these isolates with low levels of tetracycline which actives *tetM* expression as reported in other organisms (Su et al., 1992; Degrange et al., 2008). Contrasting to the *tetM* sequences, mutations in *16S rRNA* gene showed a better correlation with the observed tetracycline resistance in the *M. salivarium* isolates in this study. We believe this is the first complete documentation of a naturally occurring tetracycline resistance mechanism supported by phenotypic MIC data in a human mycoplasmal species other than the *tetM* transposon, which is fairly widespread among *M. hominis* and *Ureaplasma* spp. (Waites et al., 2014).

There are some limitations of this study. First, the number of isolates tested is small. Second, the susceptibility testing methods and MIC breakpoints have not been standardized specifically for *M. salivarium*, meaning that methods and interpretive criteria for other human mycoplasmas were utilized for reference. Third, the detailed clinical information on antimicrobial history of the patients from whom invasive isolates were obtained were not available, while the occurrence of acquired resistance could be related to their medical and antimicrobial exposure histories. Fourth, mechanisms of *tetM* regulation were not investigated in this study, e.g., no attempt to induce tetracycline resistance in susceptible isolates with *tetM* was performed.

In a summary, our study demonstrates the frequent occurrence of acquired antimicrobial resistance in *M. salivarium*, which can involve multiple drug classes, and underscores the need for culture, susceptibility testing, or a molecular testing approach to guide effective treatment.

## Data availability statement

The data presented in the study are deposited in the GenBank repository, accession numbers are: OM864597 to OM864634 for*16S rRNA* gene, ON023780 to ON023817 for *23S rRNA* gene, ON036345 to ON036382 for *rplD*, ON036383 to ON036420 for *rplV*, ON036193 to ON036230 for *gyrA*, ON036231 to ON036268 for *gyrB*, ON036269 to ON036306 for *parC* and ON036307 to ON036344 for *parE*.

## Author contributions

LX, AT, TA, and KW conceived and designed the study. DC performed antimicrobial susceptibility testing. LX performed genetic and statistical analysis. LX, AT, and KW wrote the main manuscript draft. All authors contributed to the article and approved the submitted version.

## Conflict of interest

The authors declare that the research was conducted in the absence of any commercial or financial relationships that could be construed as a potential conflict of interest.

## Publisher’s note

All claims expressed in this article are solely those of the authors and do not necessarily represent those of their affiliated organizations, or those of the publisher, the editors and the reviewers. Any product that may be evaluated in this article, or claim that may be made by its manufacturer, is not guaranteed or endorsed by the publisher.

## Abbreviations

MIC: Minimum inhibition concentration
QRDR: Quinolone resistance determining region
CLSI: Clinical and Laboratory Standards Institute
SNPs: Single nucleotide polymorphisms
