# Supplementary material for: Antimicrobial susceptibilities and mechanisms of resistance of commensal and invasive Mycoplasma salivarium isolates
Source: Front Microbiol. 2022 Aug 1;13:914464. doi: 10.3389/fmicb.2022.914464 (PMC9376445; doi:10.3389/fmicb.2022.914464)
Supplement: Supplementary file 2 [file Image_1.pdf]

Supplementary Figure 1.

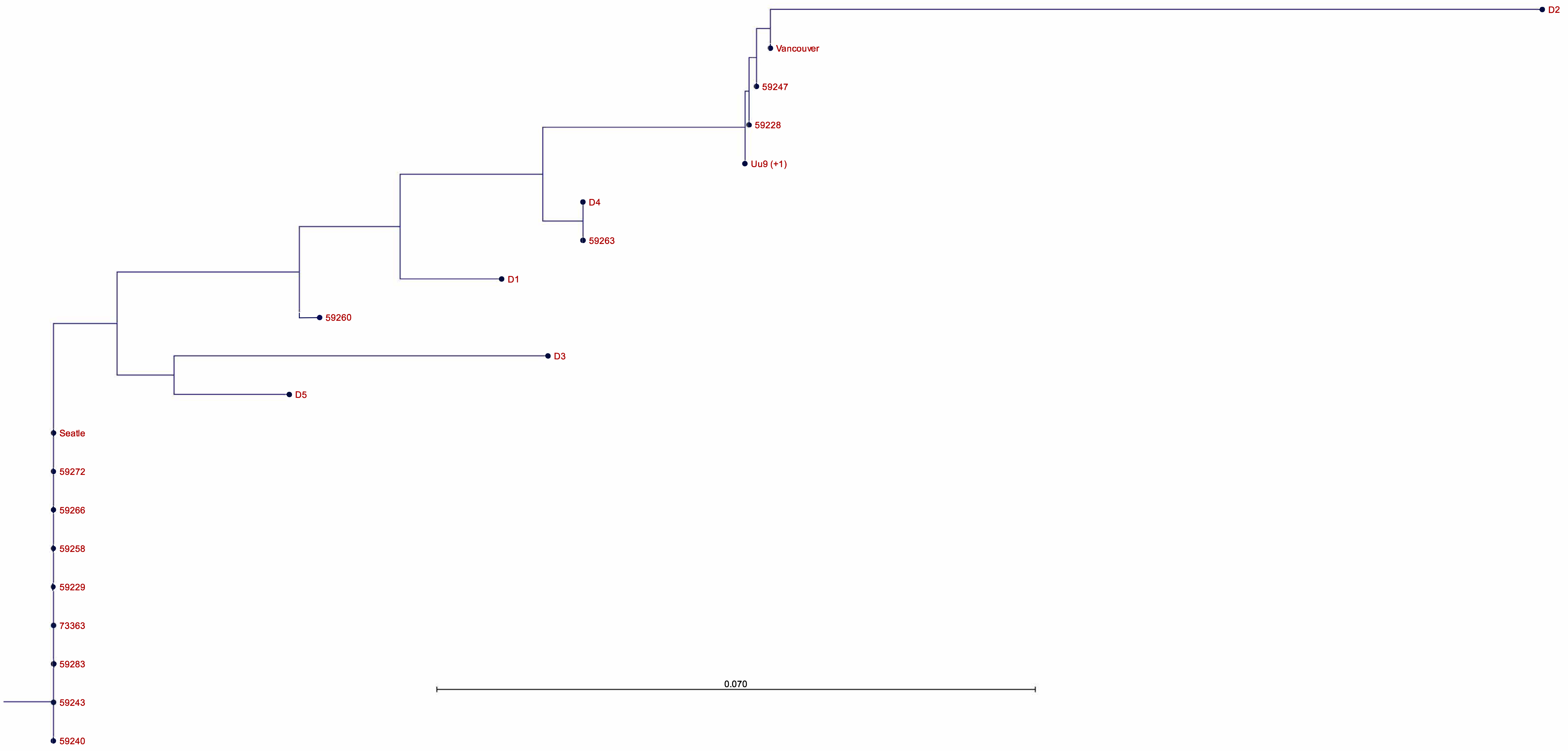

Legend of Supplementary Figure 1. Comparison of partial amino acid sequences of TetM from *M. salivarium* and *Ureaplasma* spp..obtained by Dumke. Sequences from *M. salivarium* were trimmed to have the same length of 36 amino acid as those from *Ureaplasma* spp. and alignment was performed. Phylogenetic tree was constructed based on the alignment by Neighbor Joining method using Jukes-Cantor protein distance measurement and bootstrap of 100 replicates.

Reference: Dumke R. Antimicrobial resistance in clinical isolates of *Ureaplasma* spp. from samples in Germany. Antimicrob Agents Chemother. 2021. https://doi.org/10.1128/AAC.02342-20.

Legend of Supplementary Figure 1. Comparison of partial amino acid sequences of TetM from *M. salivarium* and *Ureaplasma* spp. obtained by Dumke (Sequences named D1 to D5). Sequences from *M. salivarium* were trimmed to have the same length of 36 amino acid as those from *Ureaplasma* spp. and alignment was performed. Phylogenetic tree was constructed based on the alignment by Neighbor Joining method using Jukes-Cantor protein distance measurement and bootstrap of 100 replicates.

D1: QNCSLFPVYHGSAKSNIGIDNLIEVITNKFYSSTHR

D2: QNCSLYPLYHGSAKSNIGIEQLIEVITNKFYSSTHR

D3: HNCSLFPVYQGSAKKNIGIDNLIEVITNKFYSSTHR

D4: QNCSLFPLYHGSAKNNIGIDNLIEVITNKFYSSTHR

D5: HNCSLFPVYHGSAKSNIGIDNLIEVITNKFYSSTHR

Reference: Dumke R. Antimicrobial resistance in clinical isolates of *Ureaplasma* spp. from samples in Germany. Antimicrob Agents Chemother. 2021. https://doi.org/10.1128/AAC.02342-20.
